# Excess Wnt in neurological disease

**DOI:** 10.1042/BCJ20240265

**Published:** 2025-05-16

**Authors:** Danielle M. Pascual, Delaram Jebreili Rizi, Harsimran Kaur, Paul C. Marcogliese

**Affiliations:** 1 Department of Biochemistry and Medical Genetics, Rady Faculty of Health Sciences, University of Manitoba, Winnipeg, Manitoba, Canada; 2 Children’s Hospital Research Institute of Manitoba (CHRIM), Winnipeg, Manitoba, Canada; 3 Excellence in Neurodevelopment and Rehabilitation Research in Child Health (ENRRICH) Theme, Winnipeg, Manitoba, Canada

**Keywords:** beta-catenin, neurodegeneration, neurodevelopment, neurodevelopmental disorders, neurological disorders, Wnt proteins

## Abstract

Wnt pathways are critical developmental signaling cascades that are conserved across multicellular life. A clear role for Wnt signaling in proper neural development has been well-established, yet less is known about its sustained expression and signaling in the mature nervous system. The precise role for Wnt pathways, canonical or otherwise, and individual Wnt components (ligands, receptors, transducers, effectors, and regulators) in the mature brain are poorly understood. However, genetic evidence implicating Wnt-related components in both neurodevelopmental and neurodegenerative disorders suggests that fine-tuned regulation of Wnt signaling is required for proper nervous system development and long-term homeostasis. Much has been documented about down-regulated Wnt signaling and its association with neurological conditions. Hence, the focus of this review is to consolidate and highlight the evidence for up-regulated Wnt transcription and/or signaling in neurodevelopmental and neurodegenerative disorders with a brief discussion on the role of deregulated Wnt in cancer. Finally, we touch upon the therapeutic prospect of Wnt inhibition in the nervous system.

## Introduction

**Figure 1:**
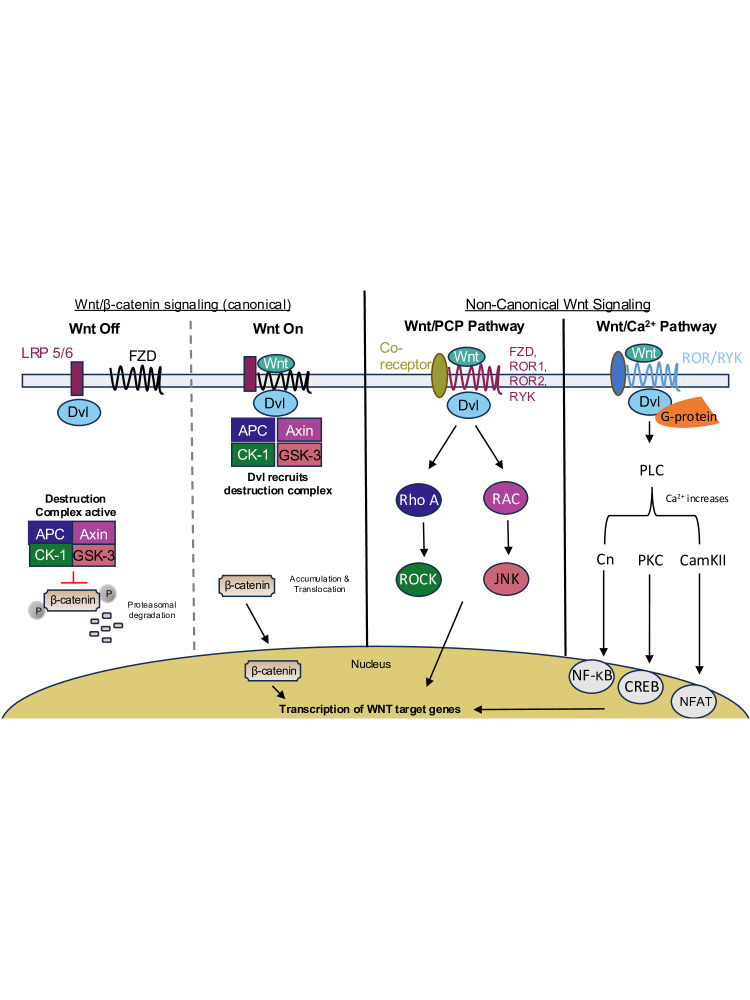
Wnt signaling pathways. In the β-catenin-dependent pathway, Wnt is considered ‘off’ when Wnt is not bound to FZD receptors and LRP5/6 co-receptors. β-catenin associates with the destruction complex (APC, Axin, CK1, GSK33), where it is phosphorylated by GSK-3 and CK1, followed by subsequent degradation via the proteasome. Wnt is considered ‘on’ when a Wnt ligand binds to LRP5/6 and FZD receptors, and the scaffold protein Dvl recruits Axin along with the kinases CK1 and GSK-3 to the membrane. This disrupts the formation of the destruction complex, preventing the phosphorylation and degradation of β-catenin. As a result, β-catenin accumulates in the cytoplasm and then translocates to the nucleus, where it activates transcription of Wnt target genes. In the Wnt/PCP pathway, small G proteins RhoA and RAC are activated via Dvl, resulting in signaling via the ROCK and Jnk signaling pathways. In the Wnt/Ca^2+^ signaling pathway, signaling is via heterotrimeric G proteins that activate PLC, which leads to a rise in intracellular Ca^2+^. This leads to the activation of calcineurin (Ca), calcium/calmodulin-dependent kinase II (CamKII), and PKC. Both CamKII and PKC activate various regulatory proteins such as NF-κB and CREB, and Cn can activate the cytoplasmic protein NFAT. CamKII, calcium/calmodulin-dependent kinase II; Wnt, wingless/integrated.

The *wingless/integrated* (*Wnt*) family of genes encode highly conserved secreted ligands that are critical for embryogenesis as well as tissue development and homeostasis [1]. These morphogens play an important role in developmental patterning and have many diverse functions in controlling proliferation, migration, differentiation, polarity, and death. The ligand was first discovered in *Drosophila melanogaster* as the *wingless* (*wg*) gene [2] and established a role in developmental patterning [3]. The name ‘Wnt’ comes from a fusion of the name of the *Drosophila* gene *wg* and the name of the vertebrate homolog, *integrated, or int-1* [4]. In mammals, 19 Wnt ligands have been identified [5]. These ligands are highly conserved across multicellular animals in both vertebrates and invertebrates. Although there is a recent appreciation for an increasing number of Wnt pathways [6], there are three main cascades: the Wnt/β-catenin-dependent, also known as the canonical pathway, the Wnt/planar cell polarity (PCP) pathway, and the Wnt/Ca^2+^ pathway (Figure 1).

### Wnt pathways

The canonical Wnt/β-catenin pathway initiates diverse signaling pathways through Wnt molecules acting as ligands that primarily interact with Frizzled (Fzd) receptors, which are seven-transmembrane G protein-coupled receptors [7]. Without active Wnt signaling, the destruction complex, composed of Axin, APC, and GSK-3, constitutively phosphorylates β-catenin, leading to its ubiquitination and proteasomal degradation. Upon secretion of Wnt from a signal-sending cell, Wnt binds Fzd and the LRP5/6 co-receptors on the signal-receiving cell. This leads to the cytosolic protein dishevelled (Dvl) becoming activated and inhibiting the destruction complex. β-catenin subsequently stabilizes, accumulates, and translocates to the nucleus, forming a transcriptional complex with Tcf./Lef family transcription factors to activate Wnt-responsive genes [8]. A growing list of regulators and interactors modulate the Wnt/β-catenin pathway. Dickkopf (DKK) inhibits Wnt signaling by binding to LRP5/6. Secreted Fzd-related proteins (SFRPs) and Wnt inhibitory factor (WIF) sequester Wnt ligands extracellularly, adding layers of regulation that define Wnt activity in a context-dependent manner pre- and post-development [9].

The Wnt/PCP pathway involves three main steps [10]. First, upstream PCP components such as Four-jointed, Dachsous, Fat, and Atrophin provide a long-range signal for global polarity direction [11]. Core PCP components such as Fzd, Dvl, Celsr, Vangl, and Prickle establish planar polarity within various cells of the body. Specifically, this is done by directing asymmetric localization at cell membranes. This co-ordination involves Fzd (the receptor for the PCP signal) and Dvl (the transducer of the signal) accumulating on one side of the cell with Vangl (membrane component) and Prickle (cytoplasmic component) on the opposite side, as Celsr facilitates intercellular communication to ensure the propagation of polarity across tissues [12,13]. PCP signaling plays a vital role in neural tube closure, co-ordinated ciliogenesis, and the organization of epithelial tissues, highlighting its importance in normal development and the maintenance of tissue structure. Some of these processes are orchestrated by downstream PCP effectors such as Daam1, Rho, Rac, Rho, JNK, and Profilin [14–17].

Finally, the Wnt/Ca^2+^ pathway was identified when injections of Wnt5a or Wnt11 mRNA into zebrafish embryos increased calcium transients in the blastodisc [18,19]. Wnt-Fzd binding activates phospholipase C (PLC) via G proteins, increasing intracellular Ca^2+^ levels and activating downstream effectors like protein kinase C (PKC). This pathway is crucial for development and is implicated in cancer [20].

To highlight the key differences between the Wnt pathways, the canonical Wnt/β-catenin pathway primarily regulates long-term processes such as gene expression, neurogenesis, cell proliferation, and synaptic stability. In contrast, the non-canonical pathways control immediate cellular responses, such as cell polarity, calcium maintenance, cell migration, cytoskeletal dynamics, and inflammation. In neurodevelopmental and neurodegenerative disorders, these pathways can interact synergistically or antagonistically. This highlights the need for fine-tuned regulation to maintain neural health [21,22].

### Wnt function

Proper Wnt signaling is essential for embryogenesis, development, cell proliferation, migration, and other processes [1]. It is also important for cell maintenance and survival [23]. Wnt is crucial for key neurodevelopmental processes such as cell proliferation, dendrite development, differentiation, migration, synaptogenesis, and axon guidance, while also maintaining synaptic plasticity and neural integrity in the mature brain [24,25]. Due to its broad role in proper brain development, when altered Wnt signaling occurs, there is a potential for disorders of the brain such as neurodevelopmental and neurodegenerative diseases.

Wnt activity varies based on temporal and cell-specific factors, such as neurons or glia, and in development or the mature nervous system [26,27]. An example of this in the context of neurodegeneration would be the hyperactivation of Wnt signaling in glial cells, which may exacerbate chronic inflammation and contribute to neuronal dysfunction in Alzheimer’s disease (AD) and frontotemporal dementia [28,29]. In comparison, in neurodevelopmental disorders (NDDs) such as autism spectrum disorder (ASD), excessive Wnt activity may disrupt synaptic pruning and lead to abnormal connectivity [30]. This variability shows the importance of tailoring therapeutic strategies to the specific context of Wnt dysregulation in each condition, as interventions should address not only the underlying molecular mechanisms but also the timing and cellular environment where Wnt signaling operates. Advances in inducible and conditional animal models, such as adult-specific knockout/knock-in systems, are critical tools for dissecting these complex dynamics.

## Wnt and brain development

Building on the diverse functions of Wnt signaling, its role in brain development highlights how tightly regulated Wnt activity is essential for processes such as neural specification, cell migration, and synaptic formation, which are critical for establishing functional neural circuits [25,31]. Canonical Wnt plays an important role in numerous homeostatic functions and is critical for early proper central nervous system (CNS) development. Haploinsufficiency of the gene *CTNNB1* that encodes β-catenin causes intellectual disability and NDDs, where β-catenin is critical for neural specification and synaptic development [32,33]. Another example of this would be the loss of function mutations in the gene *FZD3* (a gene that regulates axon guidance and neuronal migration) associated with schizophrenia [34]. Lack of *Wnt1* causes severe midbrain and cerebellar atrophy in mice [35]. Mutations in the Wnt secretion mediator wntless (*WLS*) in humans cause multiorgan defects, including microcephaly [36]. Hence, core Wnt components are critical for development, and their loss is associated with nervous system disorders.

However, what about overactivation of Wnt? The sustained expression of Wnt signaling in the mature nervous system is important for maintaining homeostasis as it regulates processes such as synaptic plasticity, neurogenesis, neuronal survival, glial cell function, and inflammation, all of which are vital for the stability and proper functioning of the nervous system [22,25]. However, proper negative regulation of Wnt is also crucial. Dkk1 knockout mice, lacking anterior head structures, demonstrate the need for Wnt inhibition during axis formation and limb morphogenesis [37]. Loss of the destruction complex member, *APC2*, leads to lissencephaly, characterized by intellectual disability and neuromotor impairments [38]. *LRP6* gene variants in infants with spina bifida showed an increase in noncanonical Wnt/PCP signaling [39]. An excess of Wnt signaling, particularly through the canonical Wnt/β-catenin pathway, can disrupt the precise cellular processes required for neural tube closure, which can cause congenital neurodevelopmental abnormalities such as neural tube defects [40,41]. The following review highlights specific examples of when the Wnt pathway is deregulated in CNS disorders, including neurodevelopmental, neurodegenerative, and neurooncological conditions. First, we will discuss excess Wnt in rare Mendelian NDDs.

### Excess Wnt in rare Mendelian NDDs

NDDs encompass a wide range of conditions that affect the growth and development of the CNS [42,43]. Within this spectrum, the literature often distinguishes between rare Mendelian subtypes and more common NDDs. Rare Mendelian conditions, such as Fragile X and Angelman syndromes, result from specific single mutations. These contrast with more prevalent clinical groups, including ASD, epilepsy, and schizophrenia, which are generally thought to have complex, heterogeneous, and polygenic inheritance patterns, although they can also be caused by single gene mutations [44]. Next-generation sequencing, such as whole-genome, exome, or long-read sequencing, has helped increase the identification of gene variants causing NDDs [45]. In both rare NDDs and common disorders of neurodevelopment such as ASD, there is an increased burden of *de novo* variants in affected individuals [46,47]. Recent advances in understanding the molecular basis of these disorders have provided renewed hope for achieving more precise and timely diagnoses and identified the genes and pathways implicated in NDDs.

Table 1 provides a list of rare NDDs associated with specific genetic variants that disrupt the Wnt signaling pathway. As mentioned above, loss of *APC2* leads to a congenital lissencephaly. In *Apc2* null mice, disrupted neuronal migration results in defects in the lamination of the cerebral cortex and cerebellum, which manifest as lissencephaly, characterized by a smooth brain surface, severe intellectual disability, seizures, and motor dysfunction [38,64]. Hence, the loss of *APC2* implicates increased Wnt/β-catenin in lissencephaly. Upon Wnt stimulation, tuberous sclerosis complex 2 (TSC2) represses β-catenin-dependent transcriptional activity. This suggests that certain phenotypic features of the TSC, such as benign tumors (hamartomas), seizures, skin abnormalities, and cognitive impairment, may arise from abnormal β-catenin signaling caused by mutations in the *TSC2* [61,62,65]. *De novo* truncations in the intronless gene interferon regulatory factor 2 binding protein-like (*IRF2BPL*) cause neurodevelopmental disorder with regression, abnormal movements, loss of speech, and seizures (NEDAMSS) [66]. Recent studies in the *Drosophila* and zebrafish CNS and patient chemically reprogrammed astrocytes show increased transcription of *WNT1* and subsequent activation of downstream Wnt signaling pathways. Moreover, pharmacological suppression of Wnt/β-catenin signaling mitigated neurobehavioral phenotypes in fruit flies and zebrafish [52]. This identified IRF2BPL as a negative regulator of Wnt/β-catenin signaling and its dysregulation in NEDAMSS. Additionally, mutations in *UBE3A*, which is a HECT domain E3 ubiquitin ligase, are linked to ASD, Angelman syndrome, and cancer. Through quantitative proteomics and reporter assays, it has been shown that a *de novo* autism-linked *UBE3A* variant p.T485A can activate Wnt/β-catenin signaling to a greater extent in comparison with the wild type [67]. Moreover, *CHD8* haploinsufficiency causes intellectual disability, ASD, and macrocephaly. *CHD8* negatively regulates β-catenin-targeted gene expression by promoting the association of β-catenin and histone H1 [49,68,69]. Moreover, pathogenic variants in *CHD2,* also known as N-cadherin, lead to a rare disease known as agenesis of the corpus callosum, cardiac, ocular, and genital syndrome [48]. Relatedly, CHD2 negatively regulates canonical Wnt signaling by forming a molecular complex with Axin and LRP5, thus leading to increased β-catenin degradation [70]. Finally, damaging variants in *PRICKLE1* are associated with progressive myoclonic epilepsy, and it has been shown to negatively regulate Wnt/β-catenin signaling through binding with Dvl and facilitating Dvl ubiquitination and subsequent degradation [53]. In sum, these examples show how deregulation of Wnt/β-catenin signaling is a likely relevant pathogenic signature of a subset of Mendelian NDDs.

**Table 1:** Mendelian neurological disorders associated with deregulated Wnt signaling.

Gene	Nervous system-related disorder	Inheritance	MIM#	Role in Wnt regulation	References
*APC2*	Cortical dysplasia, complex, with other brain malformations 10	AR	618,677	Degrades β-catenin	[38]
*CDH2*	Agenesis of corpus callosum, cardiac, ocular, and genital syndrome	AD	618,929	Interacts with axin and LRP5 increasing β-catenin degradation	[48]
*CHD8*	Autism spectrum disorder 18 (risk)	AD	615,032	Negatively regulates β-catenin gene expression	[49]
*CUL3*	Neurodevelopmental disorder with or without autism or seizures	AD	619,239	Ubiquitination of dishevelled	[50]
*FUZ*	Neural tube defects (risk)	AD	182,940	Increased Wnt/β-catenin signaling in Fuz-/- mouse embryos	[51]
*IRF2BPL*	Neurodevelopmental disorder with regression, abnormal movements, loss of speech, and seizure	AD	618,088	Ubiquitinate β-catenin, also interacts with Ck1a	[52]
*PRICKLE1*	Epilepsy, progressive myoclonic 1B	AR	612,437	Prickle-1 bound with Dvl3 and facilitated Dvl3 ubiquitination/degradation	[53]
*PRKN*	Parkinson disease, juvenile, type 2	AR	600,116	PRKN regulates beta-catenin protein levels	[54]
*PSMD12*	Stankiewicz-Isidor syndrome	AD	617,516	Up-regulation of some proteasome proteins	[55]
*RNF213*	Moyamoya disease risk	AD / AR	607,151	Ubiquitination and degradation of FLNA and NFATC2 in nooncannolcal Wnt Ca	[56]
*SCYL2*	Arthrogryposis multiplex congenita 4, neurogenic, with agenesis of the corpus callosum	AR	618,766	SCYL2 selectively induces the lysosomal degradation of Frizzled5	[57]
*SOX10*	Peripheral demyelinating neuropathy, central dysmyelination, Waardenburg syndrome, and Hirschsprung disease	AD	609,136	SOX10 competes with Tcf.4 to bind β-catenin and transrepresses its downstream target genes via its own DNA-binding	[58]
*SOX2*	Optic nerve hypoplasia and abnormalities of the central nervous system	AD	206,900	SOX2 disrupts β-catenin-Tcf./LEF mediated transcriptional activation	[59]
*TLE1*	Microcephaly and a severe neurodevelopmental disorder?	AR		Tcf. repressor	[60]
*TSC2*	Tuberous sclerosis-2	AD	613,254	Associates with GSK3 and axin and promotes β-catenin degradation	[61,62]
*WWOX*	Developmental and epileptic encephalopathy 28	AR	616,211	Preventing the nuclear import of the Dvl proteins	[63]

MIM# from Mendelian inheritance in man.

### Excess Wnt in ASD

ASD is a common condition characterized by a range of social communication deficits and restricted behaviors [71]. A notable 30–40% of individuals with ASD are comorbid with NDDs, such as seizures and motor anomalies. The etiology of ASD is complex, but twin studies and cohort studies have consistently demonstrated that genetic factors play a significant role in the etiology of ASD [72–74].

**Figure 2:**
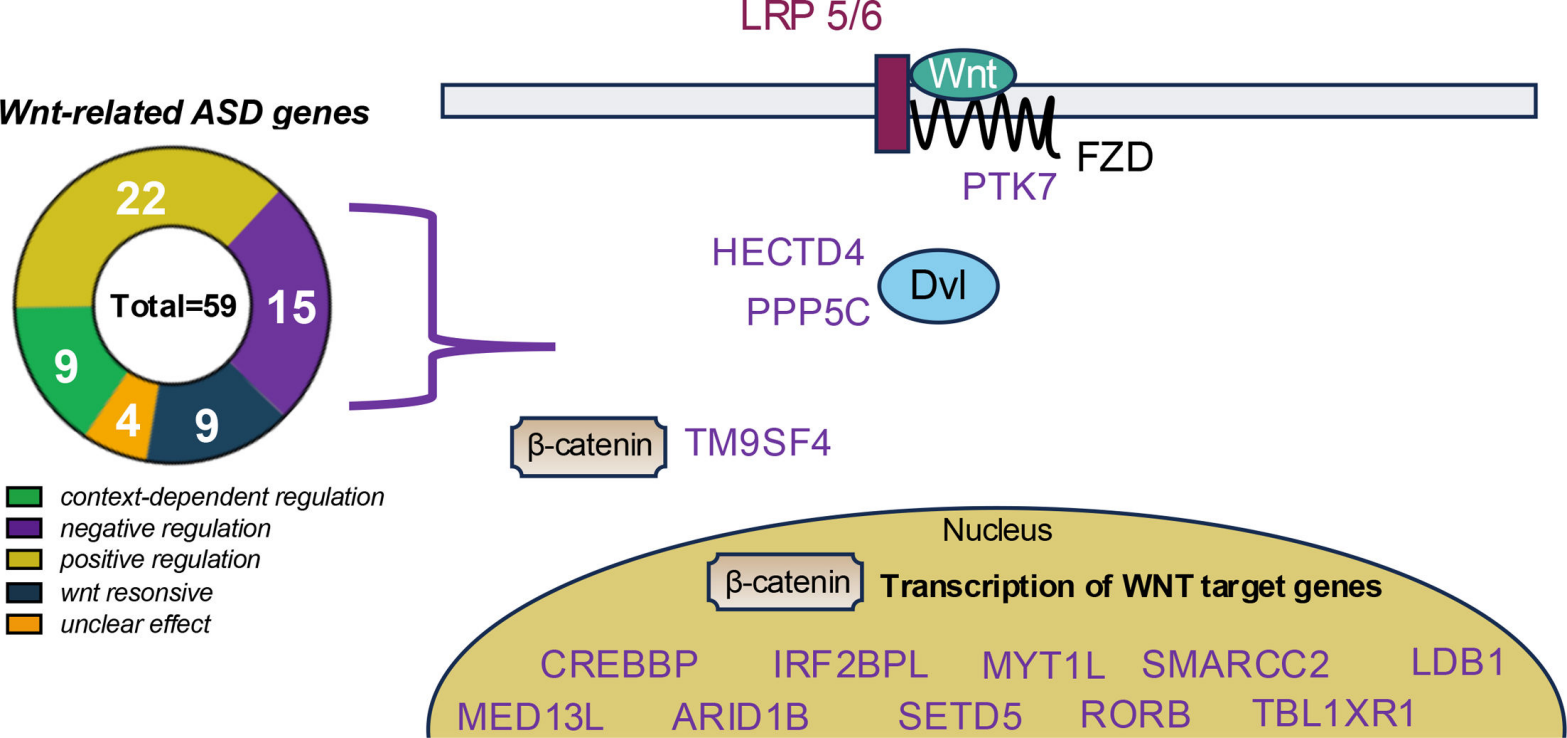
Wnt in ASD. Manually curated association of 59/102 top ASD genes and 16 negative regulators with known roles in Wnt/β-catenin signaling. ASD, Autism spectrum disorders; Wnt, wingless/integrated.

One area of significant interest in ASD research is the expression of genes associated with Wnt signaling regulation in ASD brains [75]. The convergence of gain and loss of function in Wnt/β-catenin signaling pathways in the context of ASD is particularly noteworthy. The essential function of β-catenin in brain development, growth, and the differentiation and proliferation pathways of neuronal progenitors underscores the potential role of Wnt signaling in ASD development [76]. This suggests that a wide spectrum of Wnt signaling activation may underlie the diverse manifestations of ASD. Over half of the top 102 ASD-related genes [77] (59 genes) are associated with the Wnt signaling pathway, with 18/59 acting as known negative regulators (Figure 2).

Increasing evidence suggests that dysregulated Wnt signaling plays a crucial role in the neurodevelopmental alterations observed in ASD. Several genes, including TBL1*XR1*, *MED13L*, and *PTK7* (Figure 2), provide insight into how increased Wnt/β-catenin activity may contribute to ASD pathology. TBL1XR1 is part of the NCoR/SMRT complex, which regulates Wnt/β-catenin signaling by modulating transcriptional repression, and mutations in TBL1*XR1* have been implicated in ASD and intellectual disabilities [78]. Similarly, *MED13L*, a component of the Mediator complex that regulates Wnt/β-catenin signaling [79], has been linked to ASD and intellectual disability [80]. Finally, PTK7 is a key regulator of Wnt signaling, particularly in the PCP pathway and proper embryogenesis [81] and has been associated with neural tube defects and ASD [82]. Together, these genes highlight the role of deregulated Wnt pathways in ASD and provide potential targets for further research and therapeutic intervention.

### Excess Wnt in other neuropsychiatric disorders

Major psychiatric disorders such as schizophrenia, bipolar affective disorder (BPAD), and attention-deficit hyperactivity disorder (ADHD) are common and particularly debilitating conditions. These disorders are increasingly recognized as caused by subtle dysregulations of CNS development, particularly concerning synapse formation and maintenance. However, our understanding of the biological basis for these psychiatric disorders remains limited [83]. Since Wnt signaling plays a crucial role in organizing the developing brain, it is not surprising that molecular links have tied these conditions to Wnt [84].

Schizophrenia has an estimated heritability of 65–80% and is characterized by ‘positive’ symptoms such as delusions, hallucinations, and disorganized speech, as well as ‘negative’ symptoms, including a lack of emotional affect and motivation [85]. Neuroanatomical abnormalities in schizophrenia, though subtle, include enlarged lateral ventricles, disorganized forebrain structures, loss of neuropil, and fewer synapses in pyramidal neurons [86,87]. The neurodevelopmental hypothesis suggests that these abnormalities arise from dysregulated brain development, with the Wnt signaling pathway being a key candidate for such dysregulation [88,89]. Given Wnt signaling’s critical role in brain regionalization, hippocampal development, neuronal proliferation, migration, and synapse formation, its dysregulation could lead to hippocampal volume reduction, forebrain disorganization, and synaptic defects such as those observed in schizophrenia [84]. The *WNT1* ligand has been reported to be up-regulated in individuals with schizophrenia [90], and several schizophrenia-associated genes are core components of the Wnt/β-catenin signaling pathway. Notably, a single nucleotide polymorphism (SNP) in the Wnt/β-catenin-activated transcription factor *Tcf.4* shows a significant genome-wide correlation with schizophrenia [91]. *Tcf.4* overexpression has been linked to memory impairments and deficits in prepulse inhibition, a neurophysiological correlate of schizophrenia and other psychiatric disorders [92]. Moreover, down-regulation of DKK proteins and GSK3β, both negative regulators of Wnt signaling, are genetically and pharmacologically connected to schizophrenia [93–97]. Together, a gain of Wnt/β-catenin signaling is associated with schizophrenia. However, it is known that the loss of disrupted in schizophrenia 1 (DISC1), a scaffold protein involved in synaptic development and neural migration, potentiates Wnt/β-catenin by inhibiting the destruction complex member GSK3β [98–100]. Thus, Wnt signaling loss has also been implicated in schizophrenia.

BPAD has an estimated heritability of 60–85% and is characterized by alternating phases of mania and depression [101]. Similar to schizophrenia, genomic studies have revealed disruptions in the Wnt/β-catenin signaling pathway in BPAD, implicating genes such as *DISC1*, *WNT7A*, *WNT2B*, and *Tcf.7L2* [102–107]. Additionally, SNP-based family studies have identified the Wnt/β-catenin target gene *PPARD* and the high-mobility group (HMG) box family member *HMG2L1*, the latter functioning as a negative regulator of Wnt/β-catenin signaling, as significantly associated with BPAD [107–109]. A recent genome-wide association study (GWAS) identified a noncoding SNP in the SEC14 and spectrin domain-containing 1 (*SESTD1*) locus, strongly linked to lithium-responsive BPAD. SESTD1, involved in noncanonical Wnt/PCP signaling, interacts with key proteins during neurodevelopment and in the mature CNS [107,110].

ADHD is a prevalent neurodevelopmental condition marked by symptoms of inattention, hyperactivity, and impulsivity with an estimated heritability of 77 to 88% [111,112]. ADHD affects over 5% of the global population and often coexists with other conditions, including but not limited to ASD [113]. The etiology and pathophysiology of ADHD remain largely unknown, though emerging evidence points to the interplay of genetic and environmental factors, particularly those involving the Wnt signaling pathway [114]. A recent study investigated the timing and extent of Wnt pathway alterations in ADHD across different developmental stages. Researchers performed proteomic and functional analyses on induced pluripotent stem cells (iPSCs), neural stem cells (NSCs), and differentiated neurons. Although no differences were observed at the iPSC stage, ADHD NSCs displayed altered protein expression of GSK3β and β-catenin, indicating increased Wnt activity. This heightened Wnt activity in NSCs was confirmed by reporter assays and correlated with genetic and clinical ADHD traits [114]. In addition, in the thyroid-hormone responsive-overexpressing ADHD mouse model, hippocampal proteomic analysis revealed up-regulation of *Ctnnb1* (encoding β-catenin) and other changes in Wnt-related genes, including reduced *Wnt7a* and increased Wnt inhibitors *Dkk4* and *Igfbp5* [115]. On the other hand, research has shown loss of Wnt/β-catenin signaling in immortalized patient lymphoblasts with treatment of the Wnt agonist methylphenidate [116]. Human studies have linked sex-specific genetic variations in LRP5/6 co-receptors with ADHD, further implicating Wnt signaling [117]. Additionally, in a large meta-GWAS, several genes (e.g., DUSP6, SEMA6D, ST3GAL3, FOXP1 and FOXP2, and SORCS3) linked to the Wnt pathways (canonical and non-canonical) were found to be associated with ADHD, further supporting the role of this pathway [118,119]. It is not necessarily clear from the studies above whether Wnt is lost or gained as the consequence of these genetic variations is not always known [115]. Regardless, these findings suggest that Wnt signaling dysregulation may occur at the NSC stage in ADHD, potentially contributing to the developmental deficits observed in patients. Further research into specific Wnt signaling changes in defined cell types will be critical for understanding and treating ADHD. Relatedly, *CTNNB1* is involved in multiple NDDs such as intellectual disability, schizophrenia, and ASD [32], providing further genetic evidence linking Wnt-related components across NDDs.

## Excess Wnt and neurodegenerative diseases

It has been well-established that Wnt signaling has an important role in brain development. However, in the mature CNS, it is known for its role in regeneration and plasticity, but its involvement in long-term neuronal health and neurodegenerative diseases is less clear. Importantly, all core Wnt signaling components remain expressed in the adult CNS of most animals [120]. As the brain matures and ages, the highly controlled regulation of Wnt signaling continues to exert its influence, and recent research has shed light on the pathogenesis of the following neurodegenerative disorders. Understanding how Wnt signaling contributes to the progression of these conditions can provide valuable insights into potential therapeutic interventions.

### Excess Wnt in Alzheimer’s disease

AD is the most common neurodegenerative disorder, characterized by cognitive decline, memory loss, amyloid beta (Aβ) deposition, tau aggregates, and widespread neuronal death [121]. Synapse loss is an early feature strongly associated with cognitive decline. GWAS has revealed that Wnt effectors were associated with both presenilin1 and granulin (GRN), which cause dominantly inherited forms of AD and frontotemporal dementia (FTD), respectively. Interestingly, they found that Wnt decreases GRN expression in cultured human neural progenitor cells, and knockdown of GRN resulted in an increase in Wnt expression, demonstrating the reciprocal relationship between Wnt and GRN [29]. Hence, taken together, although the down-regulation of Wnt is associated with aging and AD, there could be contexts in the CNS where deregulated Wnt contributes to neurodegeneration seen in AD and/or FTD. However, it should be noted that most evidence suggests that Wnt signaling is down-regulated in AD. Aβ may reduce Wnt signaling, contributing to synapse dysfunction and degeneration [107,108]. Studies show down-regulation of Wnt components in aging brains, with SFRP1 and Dkk1 up-regulation inhibiting Wnt pathways [122,123]. This inhibition increases Gsk3β activity and reduces β-catenin, promoting Aβ production and Tau hyperphosphorylation, both key AD features [124,125]. Thus, diminished Wnt signaling probably exacerbates AD pathology. While reduced Wnt signaling is widely implicated in aging and AD pathology, emerging evidence indicates that increased or dysregulated Wnt activity may also play a role in neurodegeneration, suggesting a more complex relationship within the CNS.

### Excess Wnt in Parkinson’s disease

Parkinson’s disease (PD) is the most common movement disorder and the second most diagnosed neurodegenerative disorder after AD, as it affects almost 1% of the population over age 60 [126]. PD motor symptoms occur when the progressive and selective degeneration of midbrain dopaminergic neurons of the substantia nigra pars compacta and their projections into the caudate putamen (striatum) result in a substantial decrease in dopamine levels. The second hallmark of PD is the accumulation of pathological α-synuclein, which is a major component of Lewy bodies and Lewy neurites present in surviving dopaminergic neurons [127].

Relating to canonical Wnt/β-catenin signaling, a main feature of PD is the regression of dopaminergic neurogenesis from neurodevelopment through aging and neurodegeneration [128,129]. It has been shown that autosomal recessive mutations in the E3 ubiquitin ligase Parkin, encoded by *PARK2,* cause early-onset PD. Moreover, Parkin represses β-catenin by inducing both β-catenin ubiquitination and degradation [54]. The loss of Parkin can lead to the accumulation of β-catenin, resulting in up-regulation in Wnt/β-catenin signaling. Moreover, autosomal-dominant mutations in LRRK2 (leucine-rich repeat kinase 2), which is encoded by *PARK8,* cause PD. Relatedly, LRRK2 is an important scaffolding protein involved in the three branches of Wnt signaling [130,131]. These studies used Lrrk2 p.G2019S-mutant knock-in mice, thought of as hyperkinase gain-of-function, and the Lrrk2 knockout mice to show that both canonical and non-canonical Wnt signaling were elevated. Hence, by examining models of familial PD, excess Wnt is implicated in the pathogenesis of this debilitating movement disorder.

### Excess Wnt in Huntington’s disease

Huntington’s disease (HD) is an autosomal dominantly inherited neurological condition that leads to involuntary choreatic movements, behavioral and psychiatric issues, and dementia [132]. It is a rare and progressive neurodegenerative disorder caused by an elongated CAG repeat *HTT* gene [133]. Multiple studies have shown that the Wnt/β-catenin pathway is altered in HD models, especially the stability and levels of β-catenin. In most cases, levels of β-catenin are increased in HD. This has been shown in multiple models, including iPSC-derived neuronal cultures [134,135], human transfected cells [136,137], mouse cell lines [138], *Drosophila* HD models [137,139], and post-mortem striatal samples from HD patients [137]. Additionally, HD neuronal cells derived from patient iPSCs have a significant dysregulation of several members of the Wnt/β-catenin signaling pathway, including the down-regulation of the components of the destruction complex and the increased expression of *Tcf.3* and *FZD* transcripts, as well as the increased expression of Wnt transcriptional targets such as *cyclin D1* (*CCND1)* [140]. This up-regulation of the gene target of the Wnt/β-catenin pathway in HD was also reproduced by other groups [135], solidifying deregulated Wnt/β-catenin signaling as an important pathogenic process in HD.

### Excess Wnt in spinocerebellar ataxia type 1

Spinocerebellar ataxia type 1 (SCA1) is a dominantly inherited neurodegenerative disease that is characterized by progressive ataxia and the degeneration of specific neuronal populations such as Purkinje cells in the cerebellum, brainstem cranial nerve nuclei, and inferior olive neurons [141]. Genetically, this disease is caused by a trinucleotide repeat expansion of a glutamine-encoding CAG tract in *ATXN1* [141]. In relation to Wnt, there are numerous genes that encode key components in the Wnt/β-catenin signaling pathway, including *Apc*, *Gsk3β*, *Ctnnb1* (encoding β-catenin), and *Lef-1*, as well as its target genes, including *Ccnd1* and *Myc*, that are expressed in adult Purkinje cells [142,143]. It has been found that ataxin-1 in a SCA1 mouse model enhances Wnt/β-catenin signaling activity at numerous levels of the pathway, prior to pathology, and that this activity increases with the size of the CAG expansion [144]. This discovery reveals an inherent pathological mechanism that enhances the activation of Wnt/β-catenin signaling in SCA1 Bergman glia (adjacent to the Purkinje cells) and likely in other cell types containing ataxin-1. Additionally, this Wnt/β-catenin enhancement potentially occurs by direct interaction with Tcf./LEF family members. Enhanced Wnt ligand secretion may also explain the dysregulation of genes associated with PCP signaling, which has been previously observed in SCA1 in previous studies [145]. Hence, the excess Wnt/β-catenin pathway is likely an important contributor to SCA1.

The above genetic evidence indicates that in sum, the complex orchestration of the availability of Wnt ligands (e.g. Wnt3a, Wnt5a) is balanced by antagonists such as DKK1 and SFRPs, which inhibit Wnt signaling. This balance is important in the prevention of both overactivation or suppression of the pathway; hence, the role of Wnt in neurodegeneration is of increasing interest [146]. The consequences of dysregulation of this organized process include cancer, neurodevelopmental, and neurodegenerative disorders. Therefore, the Wnt pathway and its players can be targeted in therapeutic strategies for various diseases, including cancer, that is discussed in the following section.

## Role of Wnt and cancer

Elevated Wnt is implicated in various cancers because it plays critical roles in regulating proliferation, death, migration, and cell fate decisions. Wnt dysregulation can affect any of these processes and lead to tumorigenesis [147]. For instance, continuous activation of β-catenin increases the proliferation of mouse neural progenitor cells [147]. Also, a proviral insertion into the *Wnt1* mouse gene causes excess transcription and can lead to mammary hyperplasia and tumors [148–150]. Both β-catenin-dependent and independent Wnt pathways play a role in embryonic neurogenesis by regulating NSC behavior during embryonic development [151]. Deficiencies or overexpression of Wnt pathways can have severe consequences, resulting in regional CNS defects or altered progenitor cell proliferation in the spinal cord and cerebral cortex [152,153].

Apart from having a role in neurogenesis, Wnt pathways are also involved in gliomagenesis and are also associated with the most malignant, common, and lethal brain cancer in adults called glioblastoma (GBM). According to the Cancer Genome Atlas classification, GBM has four classes: proneural, neural, classical, and mesenchymal [154]. Several components of multiple Wnt pathways are involved in the mesenchymal GBM subtype. These include two frizzled receptors, β-catenin, Tcf.7L1/2 and LEF1, transcriptional factors, E-cadherin, PLC gamma, calmodulins, calcineurin, and NFAT (nuclear factor of activated T cells) [155]. GBM is not associated with mutations in *CTNNB1* or *APC* but instead originates from loss/deletion (copy number loss) of FAT Atypical Cadherin 1 (*FAT1*) [156]. FAT1 is a negative effector of β-catenin, and its loss causes an overactivation of Wnt/β-catenin signaling, resulting in GBM. This is observed in 20% of GBM cases [157].

Large-scale genomic approaches have shown that GBM cells display decreased epigenetic-dependent expression of Wnt pathway inhibitors and tumor suppressors such as WIF1s, DKKs, and SFRPs [158]. These changes occur through DNA methylation and histone modification in the promoter regions [158]. These findings were significant in explaining the role of epigenetic alterations and overexpression of Wnt components, eventually leading to tumor generation. Restoring the function of these Wnt inhibitors can be a promising strategy in controlling the proliferation of GBM.

Mutations in WNT signaling genes such as *CTNNB1*, *APC*, and *AXIN1* are associated with another form of tumor found in the cerebellum called medulloblastoma (MB) [159]. β-catenin mutations in exon 3 relate to the phosphorylation site targeted by the destruction complex and result in hyperactivation of Wnt/β-catenin [158,160]. The important contrast between MB and GBM can be that most cases of MB are due to genomic mutations and not epigenetic alterations.

## Cancer and ASD

Numerous studies have found that several Wnt-related genes associated with ASD susceptibility also have associations with cancer [161]. For example, data obtained from the Brainbase database confirm that *ARID1B*, *ILF2*, and *KMT2C* are associated with glioma. What is particularly surprising about the intriguing overlap between ASD and cancer is the shared functions of these genes in chromatin remodeling and genome maintenance, as well as transcription factors [162,163]. For instance, *ADNP*, *PAX5*, *FOXP1*, *Tcf.7L2*, and *TBLXR1* are transcription factors genetically implicated in both ASD and cancer [161]. Hence, a common inference made from this finding is that since people diagnosed with ASD carry alterations in similar genes to those associated with cancer, they might have an increased risk of developing cancer. However, upon examining 702 individuals with ASD, there was no increase in childhood cancer in those with ASD [164]. This finding suggests that ASD begins to develop as a consequence of errors during fetal life at critical time periods for the proliferation of neuronal precursors, while cancer incidence is significantly correlated with age, which means errors more commonly occur during adult life in cell types susceptible to tumors [161].

Although this additional dimension adds complexity to our attempts to understand ASD, looking at mechanistic similarities can be leveraged into therapeutic strategies [165]. It may be possible to repurpose available cancer drugs with reasonable safety profiles as targeted treatments for ASD. For example, the evaluation of a rapamycin analog in patients with tuberous sclerosis included outcome measures for ASD features, along with seizures, sleep disturbances, and academic skills [161]. Therefore, ongoing research into these areas is crucial for developing a deeper understanding and more effective interventions for individuals affected by ASD. Regardless, the enrichment of ASD genes implicated in Wnt signaling points to a Wnt-related molecular signature of some forms of ASD that could inform unified diagnostic and therapeutic strategies.

## Therapeutic strategies

Since Wnt pathways are altered in many cell pathways and regulation should be tightly controlled, it may be a potential therapeutic target for many disease states. However, it is also a challenge because Wnt is so critical for multiple processes, and appropriate levels, target cells, and timing must be considered. Although both decreased and increased Wnt signaling appear to share overlapping symptoms and clinical presentations, the underlying molecular mechanisms and potential treatments will likely differ due to the distinct imbalances in Wnt activity. As a result of this complex dynamic, treatment strategies will need to be tailored specifically for different conditions involving Wnt signaling to address the specific underlying mechanisms. Some Wnt-targeting compounds are currently under evaluation in clinical trials for many neurological disorders, including PD, such as SB216763, a GSK3 inhibitor [166]. There are currently few methods for halting or slowing the progression of PD, AD, and amyotrophic lateral sclerosis (ALS) [129]. Table 2 highlights some promising compounds.

**Table 2:** Selected list of Wnt inhibitors.

Compound	Target	Inhibitor/activator of the target	Reference
LGK974	Porcupine	Inhibitor	[167]
XAV939	Tankyrase 1/Axin	Activates axin	[168]
Pyrvinium	CK1	Stabilizes CK1	[169]
NSC668036	Dsh	Inhibitor	[170]
WAY-316606	SFRP	Inhibitor	[171]
Sulindac	Dsh	Inhibitor	[172]
Curcumin	β-catenin	Inhibitor	[173]

The reason why Wnt1 specifically is targeted in therapeutics is because Wnt1 is the mediator for the communication between glial cells and Wnt1 agonists. It is responsible for providing a critical neuroprotective mechanism against oxidative stress, growth factor deprivation, and inflammation. Additionally, microglia and astrocytes are both responsible for the primary secretion of Wnt1 and Wnt1 agonists [174]. This is why in neurodevelopmental and/or neurodegenerative diseases where there is excess Wnt production, inhibitors of the Wnt pathway may have strong potential to delay and/or lessen the symptomologies of numerous neurological diseases. Many of these drugs need to be tested in respective neurodegenerative preclinical models and assessed for long-term safety and efficacy. For example, the Wnt antagonist LGK974 is currently in Phase I trials to treat solid malignancies and has also shown promising results in both the cancer field [167] and has been recently used in cultured neurons of ALS mouse models [175]. This drug is a small-molecule inhibitor of porcupine, which is a membrane-bound O-acyltransferase responsible for the secretion and palmitoylation of all Wnt ligands [176]. LGK974 is a potent down regulator of the Wnt/β-catenin signaling pathway, and further research may lead to possible therapeutics in the future for other neurological diseases that show aberrant Wnt signaling. Another Wnt inhibitor being used in the cancer field that has the potential to be used in neurological disease is the Wnt/β-catenin signaling inhibitor XAV939. This drug inhibits β-catenin translocation to the nucleus without affecting their proliferation and viability *in vitro*, as shown in LNCaP cells and CD4^+^ BRPCa lymphocytes [177]. XAV939 has also been shown to ameliorate climbing defects in the NEDAMSS disease model in *Drosophila* when the fly ortholog of *IRF2BPL* is reduced in neurons [52].

Unfortunately, these drugs are not approved and are still in clinical trials. Hence, another avenue to consider would be to use drugs and/or supplements that are readily available over-the-counter that can inhibit Wnt signaling to some degree. An example of this would be curcumin, a polyphenol that has been shown to inhibit cell growth via the Wnt/β-catenin pathway in non-small-cell lung cancer cells, resulting in decreased β-catenin [173], and is a candidate for treating neurodegenerative diseases such as PD [178]. Another example of a drug that is both readily available and has the potential to be used as a therapeutic in neurological disease is sulindac, which is primarily used as a nonsteroidal anti-inflammatory drug to treat mild-to-moderate pain. Sulindac has been shown to bind to the PDZ domain of Dvl and thereby suppress Wnt3A-induced β-catenin signaling at the level of Dvl [172]. Numerous studies demonstrate the efficacy of sulindac in models of neurodegenerative disease [179–181]. Importantly, sulindac can cross the blood–brain barrier, potentially allowing it to interact with therapeutic targets in the CNS [182].

As discussed, Wnt signaling pathways have context-dependent regulation and are involved in numerous cellular processes. It may be an ideal druggable pathway as understanding increases, since the regulation of Wnt must be tightly controlled for homeostasis. However, disrupting such a complex pathway poses a risk of harmful side effects warranting caution.

## Conclusion

Although the gain of Wnt is well-established in cancers in and outside the CNS, this review highlighted the genetic and molecular evidence for increased Wnt signaling in neurodevelopmental and neurodegenerative disorders. The role of up-regulated Wnt signaling varies significantly across neurodevelopmental, degenerative, and oncological disorders, largely depending on the timing and cell-specific context. Wnt signaling is highly context-dependent as its effects are influenced by the temporal and spatial expression patterns in specific cell types in the brain, such as neurons or glia. Although not yet fully understood, this variability emphasizes the complexity of Wnt signaling pathways and highlights the need for further research to unravel its diverse roles and implications in these conditions. Relatedly, excess Wnt in development can contribute to NDDs such as ASD and other neuropsychiatric disorders such as schizophrenia. Yet Wnt in the CNS is not only important for development but also for maintaining neuronal health and function over time. This was evidenced by deregulated Wnt in various neurodegenerative disorders, including AD, PD, HD, and SCA1. A burgeoning question is how enhanced Wnt signaling could lead to neurodegeneration or cancer, as these broad disease states have traditionally been thought of as polar in pathogenesis. Cell context is likely critical, along with the effects of deregulated Wnt in terminally differentiated cells like neurons compared with glia. Nevertheless, understanding the interplay between Wnt pathways and these conditions may lead to possible therapeutic interventions for multiple disorders.

It is important to note that Wnt signaling is a highly context-dependent pathway that plays critical roles in neural development, degeneration, and disease. Its interactions with other pathways, such as Notch [183] and Sonic Hedgehog [184], add layers of complexity that underscore its broad influence on neural processes. These interactions highlight the risk of targeting Wnt signaling therapeutically, as interfering with this cross-talk could result in unpredictable outcomes. Additionally, the context-dependent nature of Wnt signaling further complicates therapeutic targeting. Multiple variables such as ligand concentration, receptor expression, and environmental factors such as stress, inflammation, and injury can significantly influence Wnt activity [185]. These factors emphasize the need for patient-specific approaches in developing Wnt-targeted therapies, as a one-size-fits-all strategy is unlikely to be effective. In the mature brain, the role of Wnt signaling is especially challenging to study compared with its role in development. Unlike the relatively well-defined processes of development, Wnt signaling in the adult brain is more subtle and complex, with cell-specific interactions between Wnt ligands, receptors, and downstream signaling pathways that are harder to pinpoint [186]. This complexity necessitates advanced tools and research, such as conditional animal models, including adult-specific knockout systems and inducible mouse models. These tools are critical for dissecting the precise roles of Wnt signaling in the adult brain and for understanding how its dysregulation contributes to neurodegeneration and other disorders.

## Abbreviations

AD: Alzheimer’s disease
ADHD: attention deficit hyperactivity disorder
ALS: amyotrophic lateral sclerosis
ASD: autism spectrum disorders
Aβ: amyloid beta
BPAD: bipolar affective disorder
CCND1: cyclin D1
CNS: central nervous system
CamKII: calcium/calmodulin-dependent kinase II
DISC1: disrupted in schizophrenia 1
DKK: dickkopf
DNVs: *de novo* coding variants
DVL: dishevelled
FAT1: FAT atypical cadherin 1
FTD: frontotemporal dementia
Fzd: frizzled
GBM: glioblastoma
GRN: granulin
HD: Huntington's disease
HMG: high mobility group
IRF2BPL: interferon regulatory factor 2 binding protein-like
LRRK2: leucine-rich repeat kinase 2
MB: medulloblastoma
NDDs: neurodevelopmental disorders
NEDAMSS: neurodevelopmental disorder with regression, abnormal movements, loss of speech, and seizures
NFAT: nuclear factor of activated T cells
NSCs: neural stem cells
PCP: planar cell polarity
PD: Parkinson’s disease
PKC: protein kinase C
SCA1: spinocerebellar ataxia type 1
SESTD1: SEC14 and spectrin domain-containing 1
SFRPs: secreted Fzd-related proteins
SNP: single nucleotide polymorphism
TSC2: tuberous sclerosis complex 2
WIF, WLS: wntless,Wnt inhibitory factor
Wnt: wingless/integrated
iPSCs: induced pluripotent stem cells
